# Improved Speech in Noise Perception in the Elderly After 6 Months of Musical Instruction

**DOI:** 10.3389/fnins.2021.696240

**Published:** 2021-07-09

**Authors:** Florian Worschech, Damien Marie, Kristin Jünemann, Christopher Sinke, Tillmann H. C. Krüger, Michael Großbach, Daniel S. Scholz, Laura Abdili, Matthias Kliegel, Clara E. James, Eckart Altenmüller

**Affiliations:** ^1^Institute for Music Physiology and Musicians’ Medicine, Hanover University of Music, Drama and Media, Hanover, Germany; ^2^Center for Systems Neuroscience, Hanover, Germany; ^3^Geneva Musical Minds Lab, Geneva School of Health Sciences, University of Applied Sciences and Arts Western Switzerland (HES-SO), Geneva, Switzerland; ^4^Faculty of Psychology and Educational Sciences, University of Geneva, Geneva, Switzerland; ^5^Division of Clinical Psychology and Sexual Medicine, Department of Psychiatry, Social Psychiatry and Psychotherapy, Hanover Medical School, Hanover, Germany; ^6^Center for the Interdisciplinary Study of Gerontology and Vulnerability, University of Geneva, Geneva, Switzerland

**Keywords:** speech in noise, musical training, speech processing, hearing, auditory functioning, elderly

## Abstract

Understanding speech in background noise poses a challenge in daily communication, which is a particular problem among the elderly. Although musical expertise has often been suggested to be a contributor to speech intelligibility, the associations are mostly correlative. In the present multisite study conducted in Germany and Switzerland, 156 healthy, normal-hearing elderly were randomly assigned to either piano playing or music listening/musical culture groups. The speech reception threshold was assessed using the International Matrix Test before and after a 6 month intervention. Bayesian multilevel modeling revealed an improvement of both groups over time under binaural conditions. Additionally, the speech reception threshold of the piano group decreased during stimuli presentation to the left ear. A right ear improvement only occurred in the German piano group. Furthermore, improvements were predominantly found in women. These findings are discussed in the light of current neuroscientific theories on hemispheric lateralization and biological sex differences. The study indicates a positive transfer from musical training to speech processing, probably supported by the enhancement of auditory processing and improvement of general cognitive functions.

## Introduction

A vast part of our daily communication is embedded in background noise. This poses a challenge in understanding speech, which is a frequently stated problem among the elderly (Pichora-Fuller, 1997; Anderson et al., 2011). Communication difficulties may have profound consequences for quality of life (Ciorba et al., 2012) and thus present an increasingly important public health problem. Understanding speech in noise (SIN) is a complex skill that is subject to a fairly large age-related decline (Anderson et al., 2011). This loss is not merely attributable to a degradation of structures of the auditory periphery; more and more central and cognitive domains are shown to contribute to SIN (Nahum et al., 2008; Parbery-Clark et al., 2009; Wong et al., 2009; Strait and Kraus, 2011; Moore et al., 2014; Ross et al., 2020). A fundamental mechanism for perceiving SIN involves the transformation of a complex acoustic environment into the representation of diverse auditory objects. This so-called “auditory stream segregation” is a major part of a process which has been named “auditory scene analysis” by Bregman (1990). The formation of an auditory object is mainly determined by the location, timing and pitch of the auditory stimulus (Anderson and Kraus, 2010).

The processing steps of SIN are heavily interwoven and localized within all levels of the auditory pathway (see also “reverse hierarchy theory” in Nahum et al., 2008). Beside the utilization of low-level information and bottom-up processing, effective top-down control of early auditory stages can be assumed as well, exerted via, for example, corticofugal pathways and the medial olivocochlear bundle (de Boer and Thornton, 2008; de Boer et al., 2012). Thus, stream segregation could be mediated by cognitive processes such as working memory (Parbery-Clark et al., 2009, 2011), inhibition (Ross et al., 2020), and attention (Wong et al., 2009; Strait and Kraus, 2011; Zendel et al., 2019), which seem to be conducive to SIN. In addition, it seems likely, that the implication of cognitive functions becomes particularly important with increasing age (Zendel and Alain, 2013). This was investigated in a functional magnetic resonance imaging (fMRI) study while performing SIN tasks (Wong et al., 2009). The researchers found stronger recruitment of attention- and working memory-related cortical areas in older compared to younger subjects, while activation of auditory regions was reduced. This finding supports the “decline-compensation hypothesis,” which assumes that a decline in sensory processing is compensated for by stronger recruitment of more general cognitive domains (Wong et al., 2009).

Since the initial report by Parbery-Clark et al. (2009), a growing body of evidence showed an advantage of SIN performance of musicians over non-musicians (Parbery-Clark et al., 2011; Strait and Kraus, 2011; Zendel and Alain, 2012; Slater et al., 2015; but see Ruggles et al., 2014; Boebinger et al., 2015). However, existing data do not necessarily help to identify the exact underlying mechanism by which musical training may enhance SIN. Because a multitude of cues can be utilized in order to solve SIN tasks, and most of these cues are required to be used when music making, it is not yet clear which ones are responsible for the musicians’ advantages in SIN (for a review see Coffey et al., 2017).

According to Thorndike and Woodworth’s (1901) early conclusion, successful transfer necessitates a certain degree of “overlap” between the trained and the transfer skill. This makes a generalization of training effects to untrained tasks unlikely when the latter are very dissimilar from the trained task. Until today this conception of overlap or similarity as a necessity for transfer applies and is part of many current theories (Barnett and Ceci, 2002; Patel, 2011). One of many commonalities of music and speech is their use of rhythm and pitch for conveying information (Kraus and Chandrasekaran, 2010), that is the temporal and spectral organization of sound. Therefore, during music listening auditory patterns may be identified and enable the formation of meaningful elements (for example, segregating a melody or single instruments out of a musical piece). This is also an essential component of SIN performance, where spoken words have to be recognized out of a competent noisy auditory stream. The importance of spectral information in recognizing speech was confirmed in a recent experiment. Using a longitudinal design, Dubinsky et al. (2019) showed that improvements of SIN after choir singing were fully mediated by improvements in pitch discrimination.

There is a vast body of literature showing beneficial auditory processing in connection to musical activities or speech listening interventions (Kraus and Chandrasekaran, 2010; Särkämö et al., 2010; Zendel and Alain, 2012). The OPERA hypothesis (Patel, 2011) aims to predict the success of music-driven adaptive plasticity within speech-processing networks. According to the model, five conditions must be met by musical training to achieve successful transfer: an *overlap* of brain networks involved in processing speech and music; *precise* encoding of acoustic features; *emotionality*; *repetitive* musical activity and *attentive* practice. Since all of these conditions are frequently met in musical activities, the notion that SIN benefits from musical instruction is plausible. To prove this hypothesis and rule out potential underlying confounders randomized controlled trials (RCTs) were conducted. The first longitudinal evidence comes from a study with children showing beneficial effects of music intervention on SIN after 2 years of training (Slater et al., 2015). This result was confirmed in children (Lo et al., 2020) and older adults with sensorineural hearing loss (Dubinsky et al., 2019) as well as normal hearing adults (Fleming et al., 2019; Zendel et al., 2019). However, all above studies had at least some methodological flaws (e.g., unbalanced sex ratios and small sample sizes) which limit generalizability. In order to corroborate these promising results and to test if positive effects also emerge during perceptive musical training, we conducted a study of 156 participants in which playing piano was compared to music listening/musical culture groups.

## Materials and Methods

### Subjects

In the present RCT 156 subjects (females = 92, males = 64) from Hanover (Germany; *N* = 92) and Geneva (Switzerland; *N* = 64) between 62–78 years of age (mean = 69.7, *SD* = 3.5) participated. Most participants were recruited in response to local newspaper advertisements (74%) or heard about the study from others (16%). Detailed demographic information is given in Table 1. All subjects were right-handed (Oldfield, 1971), retired, non-reliant on hearing aids and did not report any neurological, psychological or severe physical health impairments. Before inclusion they were screened for global cognitive functioning using the Cognitive Telephone Screening Instrument (COGTEL; Kliegel et al., 2007; Ihle et al., 2017). The test battery was administered in a face-to-face fashion and assessed performance of six cognitive domains (verbal short- and long-term memory, working memory, verbal fluency, inductive reasoning and prospective memory). All subjects achieved total scores (all > 15) well-above an *a priori* defined threshold (= 10) excluding people with beginning or advanced dementia. The cut-off value at 10 was empirically determined on the basis of the original publication (Kliegel et al., 2007) to represent the threshold for participants that were below 2 SDs of the original validation sample. Participants were screened for depression with the 15-item Geriatric Depression Scale (Sheikh and Yesavage, 1986) and excluded with scores > 8 (mild–moderate depression). Importantly, all participants were non-musicians who had less than 6 months of regular musical practice over their lifespan. As an additional inclusion criterion, all participants had to give their consent to accept to become randomly assigned to one of the intervention groups and not to participate in any other musical course during the study. At the same time we emphasized that the study aims were to compare two distinct music interventions and that both may have positive impact on cognitive functioning. The experiment was conducted in accordance with the declaration of Helsinki and approved by local ethics committees. All participants gave written consent to participate and were free to withdraw from the study at any time.

**TABLE 1:** Demographic information of the sample.

	Geneva	Hanover	Total
N	64	92	156
Age (SD)	70.23 (3.65)	69.30 (3.34)	69.69 (3.49)
Male/Female (%)	14/50 (22/78)	50/42 (54/46)	64/92 (41/59)
Income (SD)	2.97 (1.00)	2.74 (0.94)	2.83 (0.97)
Education (SD)	3.48 (1.18)	4.18 (1.41)	3.90 (1.36)
COGTEL (SD)	30.50 (7.14)	32.38 (7.25)	31.61 (7.24)

*Income and Education level from 1 to 5 (<25, 25–75, 75–125, 125–175, >175% of national average) and 1–6 (elementary school, middle school, high school, Bachelor, Master, PhD), respectively, with higher scores indicating higher socioeconomic status.*

### Intervention

The present study is part of an extensive investigation (“Train the brain with music”) aiming to shed light on the effects of music on cognition and the brain in the elderly (for full protocol see James et al., 2020). Randomized allocation to Playing Piano (PP; *N* = 74) or Musical Culture (MC; *N* = 82) groups was stratified to ensure groups were matched in age, sex, cognitive functioning (total score of the COGTEL) and education level. The allocation was concealed to participants until individual baseline measurements were completed. Participants in both groups attended weekly 60 min sessions, which were administered in participant-dyads for PP and in small groups of 4–7 subjects for MC. 19 PP and 7 MC teachers were recruited from local music universities. Most teachers were enrolled in a musical performance and education course (*N* = 21) with piano (*N* = 16; all PP teachers) or a different main instrument (*N* = 5; all MC teachers). The remaining 5 teachers were studying music education (*N* = 3; 1 MC and 2 PP teachers) or music theory (*N* = 2; 1 MC and 1 PP teacher). All possessed at least a Bachelor’s degree and had several years of teaching experience. Throughout the study, all teachers were supervised by university-level professors of music education and piano pedagogy. During supervisory meetings, the teachers had the opportunity to exchange experiences, discuss ideas and report the progress of their group(s). In order to check that the teaching quality was adequate, each group was visited and rated at least once by one of the co-authors (D.S.S., C.E.J., E.A.) during the 6-month intervention period.

The teachers accompanied their students over the entire period with the exception of two participants of PP, where a group was recomposited due to divergent progress. PP sessions took a sensorimotor-based “bodily-holistic” approach to piano education, involving clapping and walking to a beat and free exploration of the full range of the keyboard, in addition to more traditional listen-and-repeat exercises and improvisations on the instrument. Music reading was introduced with an approach specially developed for older people based on Schlichting’s “Piano Prima Vista” (Inter-Note GmbH Musikverlag 2013) and the Hall Leonard piano method for adults (ISBN 9789043134378). PP participants learned to play simple musical pieces using different textbooks, for example “A Dozen a Day” vol. 1 (ISBN 9780711954311) or “Jugend-Album für Klavier” by Schmitz (ISBN 9783932587412). MC sessions emphasized analytic listening and experiencing, understanding and appreciating music through discussion of a variety of musical aspects, for example musical genres, instrument groups, music history and famous composers, but also some music theory (e.g., Sonata form; for more details, see James et al., 2020). Active music-making, however, was avoided. The last 10 min of the two courses were used to explain the homework to be done for the coming week. For PP, this also included practice strategies.

While we developed a guideline with topics for MC, only the first three sessions were completely standardized in PP. The content of the following lessons were deliberately not specified in detail, as we expected large variability in the musical abilities, learning progress and needs of our older participants. However, the basic principles were maintained in all groups throughout the course in order to offer systematic piano lessons. This included the use of the material provided, physical warm-up, listening to the sound, bimanual coordination and, to a lesser extent, reading music scores (more details can be found in Supplementary Material). Individual wishes, experiences and interests of the participants were also taken into account in MC. This required a high degree of adaptability on the part of the teachers, but in return enabled highly individualized and joyful music lessons.

Participants were asked to commit to attending at least 20 sessions within 6 months and complete the assigned homework for ∼30 min/day. To this end, each PP participant received an electronic piano (Yamaha P-45) with headphones (Yamaha HPH-50) and a piano stool. After 6 months, the subjects were asked about how much time they spent doing their homework during the last 3 months.

### Speech in Noise

The German (Wagener et al., 1999b) and French (Jansen et al., 2012) versions of the International Matrix Test were used for the Hanover and the Geneva participants, respectively. During this test, participants listened to 20 short and syntactically easy sentences presented via audiometric headphones (Sennheiser HDA 300). After each sentence the participants had to repeat all words they understood. All sentences had the same syntax of five words (name, verb, number, adjective, noun) without semantic cues (e.g., *Peter got three large stones*). The Matrix Test uses an adaptive procedure (determined using a maximum likelihood estimator) with variable step sizes. It aims to identify the 50% threshold of SIN, the so-called “Speech Reception Threshold” (SRT). The SRT indicates the signal-to-noise ratio (SNR) at which 50% of the presented words are correctly understood. During the developmental process of the Matrix Test, 100 different sentences were recorded with a sampling rate of 44,100 Hz and a resolution of 16 bits (Wagener et al., 1999b; Jansen et al., 2012). After equalizing the level on the basis of the root-mean-square, the sentences were segmented into single words. Finally, the words were recombined into new sentences under consideration of the coarticulation of the adjacent word. The speech rate was 3.9 and 4.2 syllables/sec in the German and French version, respectively (Wagener et al., 1999b; Jansen et al., 2012). The background noise consisted of the same long-term spectrum as the speech, which yielded an optimal spectral masking (Wagener et al., 2003). Therefore, random sequences of the entire speech material were superimposed 30 times in order to provide a stationary noise without strong fluctuations. During the testing procedure, the level of the speech-shaped background noise was kept constant at 65 dB SPL, while the speech level changed as a function of the participant’s performance. If the subject repeated at least three words correctly, the speech level of the next presentation was reduced; else the speech level was increased. The initial sentence was presented at a SNR of 0 dB (background noise and speech both 65 dB). In total, four conditions with different sets of sentences were performed. First, in order to familiarize the participants with the task and to reduce training effects (Wagener et al., 1999a) stimuli were presented binaurally without background noise, providing an intelligibility score (percentage of words perceived correctly without noise). After that, the sentences were monaurally presented with background noise, starting with a random side. The last condition comprised a binaural presentation with noise. Each block was conducted with test lists of 20 sentences. We randomized the presentation of the stimuli over all time points so that the participants did not hear the same set of sentences twice. In the German version a male speaker was chosen, whereas in the French version a female speaker was selected as no male speaker was available. Both sites used the same audio interface (ESI Maya 22).

### Data Analysis

Data were analyzed in a Bayesian multilevel approach using the package brms (Bürkner, 2017, 2018) in R (R Core Team (RCT), 2020). The Markov chain Monte Carlo (MCMC) estimation approach was applied with four chains. Iterations were set to 4,000–6,000 with a warm-up of half the number of iterations. Adapt_delta was kept between 0.80 and 0.97 and max_treedepth was set to 10. Both parameters are algorithm-specific tuning tools. Adapt_delta indicates the target acceptance rate during the adaptation phase of the Markov chains and aims to solve divergence problems. Max_treedepth indicates the maximum number of steps each iteration may take and aims to tackle efficiency problems. Independent models for each condition were established. The dummy-coded (0|1) variables Time (Baseline| 6 months), Group (MC| PP), Sex (Female| Male) and Site (Hanover| Geneva) as well as the continuous variables COGTEL, Intelligibility, Age and an additional Time-by-Group interaction were selected as predictors, centered and modeled with varying intercepts and slopes for each participant nested in Site. Three additional models were built aiming to address a possible different progress of SIN in men and women. Therefore, the Time-Group interaction was expanded by a third variable Sex. A last model was computed retrospectively for right SIN only, testing a hypothesized Time-by-Group-by-Site interaction. Expected log predictive densities (ELPD) were estimated by leave-one-out cross-validation (LOO; Vehtari et al., 2017) and applied in order to compare models. For the distribution of the response variable the Gaussian family was selected. Normally distributed weakly informative priors were applied. All models converged without problems as indicated by Rhat values ≤ 1.01 and generated visually well-mixed chains. As a measure of fit a Bayesian version of R^2^ was applied using the bayes_R2 method (Gelman et al., 2018).

For analyzing the correlation among SIN conditions Pearson’s product-moment correlation coefficient was used. The analysis included the first measurement time point and was performed for both sites separately.

## Results

*N* = 10 participants (6.4%; 2 PP, 8 MC; 4 Male, 6 Female) dropped out during the 6-month intervention period for reasons of time (3), health (2) and/or family (1), lack of interest (3) or stress (1). Two-sided *t*-test revealed no significant difference in time spent for daily homework between PP (40.4 min, *SD* = 22.6) and MC (36.5 min, *SD* = 22.16), *t*(143.6) = −1.06, *p* = 0.29). Population-level effects of each model are given in Table 2. All three models revealed a strong site-effect with higher SRTs in the Geneva sample. The estimated site-difference ranged from 1.85 dB, 95% credible interval^1^ (CI) [1.41, 2.27] for the left ear to 2.30 dB [1.96, 2.64] binaurally. We considered differences between groups or conditions most likely to be real if the CI did not overlap zero. In all conditions a beneficial influence of Intelligibility (binaural speech understanding without noise) on SIN could be shown (binaural: −0.11 dB [−0.14, −0.08]; left: −0.19 dB [−0.22, −0.15]; right: −0.09 dB [−0.16, −0.02]). Age was negatively associated with SIN indicating a yearly loss of 0.07 dB [0.02, 0.12] for the left and up to 0.13 dB [0.02, 0.23] for the right ear. No model revealed clear effects of Group or COGTEL.

**TABLE 2:** Population-level effects of the two-way-interaction models.

	Binaural SIN			Left SIN			Right SIN		
	Estimate	l–95% CI	u–95% CI	Estimate	l–95% CI	u–95% CI	Estimate	l–95% CI	u–95% CI
**Intercept**	–6.70	–6.86	–6.56	–3.70	–3.88	–3.52	–3.67	–4.09	–3.27
**Time**	–0.14	–0.29	0.00	–0.25	–0.46	–0.04	–0.09	–0.26	0.08
**Group**	0.00	–0.29	0.30	–0.02	–0.37	0.34	0.38	–0.38	1.10
**Sex**	0.34	0.02	0.65	0.44	0.05	0.84	0.09	–0.73	0.87
**Site**	2.30	1.96	2.64	1.85	1.41	2.27	2.05	1.08	2.95
**COGTEL**	–0.01	–0.03	0.01	–0.01	–0.04	0.02	–0.04	–0.10	0.02
**Intelligibility**	–0.11	–0.14	–0.08	–0.19	–0.22	–0.15	–0.09	–0.16	–0.02
**Age**	0.10	0.05	0.14	0.07	0.02	0.12	0.13	0.02	0.23
**Time:Group**	0.08	–0.21	0.36	–0.41	–0.81	–0.01	–0.01	–0.34	0.33
**R^2^ (Error)**	0.91 (0.01)			0.82 (0.02)			0.95 (0.01)		

*Upper and lower CIs are provided for each estimate.*

### Binaural SIN

**FIGURE 1:**
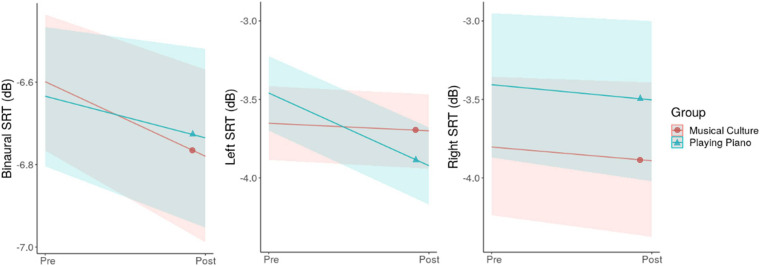
Time-by-Group interaction plots for binaural, left and right SRT. The solid lines represent the estimated averages and the shaded areas their CIs.

Modeling binaural SIN revealed a beneficial effect of Time (−0.14 dB [−0.29, 0.00]) and an influence of Sex, with men showing a 0.34 dB [0.02, 0.65] higher SRT than women. No Time-by-Group interaction occurred. Both groups improved their SRT by an average of −0.14 dB (Figure 1, left).

### Left SIN

The model for left SIN also showed an improvement over Time (−0.25 dB [−0.46, −0.04]) and a disadvantage for men compared to women (0.44 dB [0.05, 0.84]. Additionally, a negative Time-Group interaction manifested suggesting that the improvement only applies to PP (−0.41 dB [−0.81, −0.01]; Figure 1, middle). On average, PP improved its left SRT by −0.46 dB.

### Right SIN

In contrast to the former models, for the right SIN no effects of Time or Sex could be detected, nor a Time-by-Group interaction (Figure 1, right).

Because effects of sex were found for binaural and left auditory presentations, three additional models were built aiming to address a possible different progress of SIN in men and women. Therefore, we expanded the Time-Group interaction by a third variable Sex. In comparison to their corresponding two-way interaction models, the expanded models show a better fit (ELPDs) and smaller standard errors (SEs) for monaural conditions indicated by their difference (delta scores; left: ΔELPD = 2.6, ΔSE = −3.2; right: ΔELPD = 5.3, ΔSE = −5.0) and a poorer fit for the binaural condition (ΔELPD = −3.5, ΔSE = 5.4). The estimates of the population-level effects for each three-way interaction model are given in Table 3.

**TABLE 3:** Population-level effects of the three-way-interaction models.

	Binaural SIN			Left SIN			Right SIN		
	Estimate	l–95% CI	u–95% CI	Estimate	l–95% CI	u–95% CI	Estimate	l–95% CI	u–95% CI
**Intercept**	–6.70	–6.86	–6.55	–3.70	–3.88	–3.52	–3.67	–4.09	–3.25
**Time**	–0.14	–0.29	0.01	–0.25	–0.45	–0.03	–0.09	–0.26	0.08
**Group**	0.00	–0.30	0.29	–0.01	–0.37	0.35	0.37	–0.40	1.12
**Sex**	0.44	0.10	0.76	0.45	0.08	0.84	0.08	–0.71	0.87
**Site**	2.30	1.96	2.64	1.85	1.43	2.26	1.99	1.03	2.94
**COGTEL**	–0.01	–0.03	0.01	–0.01	–0.04	0.02	–0.04	–0.09	0.02
**Intelligibility**	–0.10	–0.14	–0.08	–0.19	–0.22	–0.15	–0.09	–0.16	–0.02
**Age**	0.10	0.05	0.14	0.07	0.02	0.12	0.13	0.03	0.23
**Time:Group**	0.07	–0.22	0.37	–0.42	–0.84	–0.02	–0.02	–0.34	0.31
**Time:Sex**	0.32	0.03	0.61	0.47	0.05	0.89	0.26	–0.08	0.60
**Time:Group:Sex**	0.05	–0.52	0.61	0.17	–0.59	0.96	–0.24	–0.88	0.40
**R^2^ (Error)**	0.91 (0.01)			0.82 (0.02)			0.95 (0.01)		

As the estimates did not differ substantially from the former models (compare with Table 2) only the Time-by-Sex and Time-by-Group-by-Sex interactions are discussed below.

**FIGURE 2:**
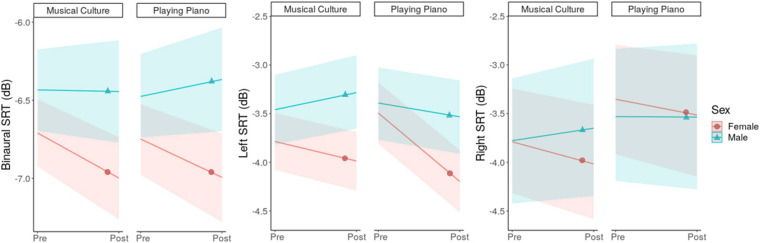
Time-by-Group-by-Sex interaction plots for binaural, left and right SRT. The solid lines represent the estimated averages and the shaded areas their CIs.

Time-Sex interactions indicate disadvantageous effects for men in comparison to women across binaural (0.32 dB [0.03, 0.61]) and left (0.47 dB [0.05, 0.89]) SIN conditions, but potentially not for the right side (0.26 dB [−0.08; 0.60]; Figure 2). Only if men attended to piano lessons they improved in left SIN (−0.28 dB; women −0.70 dB). In all other conditions, however, they did not change or worsened. Whether the interactions are dependent on group membership is inconclusive, as the large CIs of the three-way-interactions indicate.

A further model was computed retrospectively for right SIN only, testing a hypothesized Time-by-Group-by-Site interaction. The effect of group over time was different among both sites. While PP in Hanover showed an improvement, Swiss PP worsened slightly (0.67 dB [0.05, 1.31]). This model yielded a better fit than its two-way interaction counterpart (ΔELPD = 5.7, ΔSE = −6.3).

Using Pearson’s r we found different correlations among SIN conditions between both sites (Table 4). While in the Hanover sample a moderate correlation between left and right side was found (*r* = 0.41, *p* < 0.001), the correlation was not significant in the Geneva sample. Similarly, the correlation between right and binaural SIN was 0.18 points higher in Hanover (*r* = 0.57, *p* < 0.001) compared to Geneva (*r* = 0.39, *p* = 0.001). On the other hand, a very strong correlation could be detected between the left and the binaural condition in Geneva (*r* = 0.87, *p* < 0.001) which was 0.20 points lower in Hanover (*r* = 0.67, *p* < 0.001).

**TABLE 4:** Correlation matrix of monaural and binaural conditions of SIN.

	Hanover			Geneva		
	Left	Right	Binaural	Left	Right	Binaural
**Left**						
**Right**	*r* = 0.41 (*p* < 0.001)			*r* = 0.20 (*p* = 0.114)		
**Binaural**	*r* = 0.67 (*p* < 0.001)	*r* = 0.57 (*p* < 0.001)		*r* = 0.87 (*p* < 0.001)	*r* = 0.39 (*p* = 0.001)	

## Discussion

The results of the present study show that after 6 months of musical training, binaural SRTs improved in both groups by an average of −0.14 dB (Figure 1, left). Additionally, PP improved their left SRT by −0.46 dB, while MC showed no change over time (Figure 1, middle). When considering the influence of the participants’ sex, beneficial effects were almost exclusively present in women. For example, women improved their binaural SRT by −0.30 dB, whereas men’s binaural SRT did not substantially change (+0.02 dB; Figure 2, left). The only exception was the improvement in left SRT where both men and women of PP benefited. Here we could estimate a SRT improvement of −0.70 and −0.28 dB in women and men, respectively (Figure 2, middle). In comparison to the outcomes of other longitudinal studies our effects are relatively small and may not be of clinical significance: Slater et al. (2015) showed improvements of −2.1 dB after 2 years of musical training but no significant improvements after 1 year. And Lo et al. (2020) revealed a SRT lowering by 1.1 dB after only 12 weeks of musical training. A likely explanation for this difference resides in the age-related decline in neuroplasticity (Park, 2013). Both mentioned studies were in children, and the present study investigated music-driven effects on SIN in the elderly. Our results come closer to the findings of Dubinsky et al. (2019), who showed an improvement of −0.81 dB in elderly following 10 weeks of choir singing. But due to their very unbalanced sex-ratio with 91% women, one should be careful with a generalization of the findings (see last discussion point). Furthermore, in our study, we excluded participants who had hearing problems or were dependent on hearing aids. Since the greatest gains in auditory tasks have been shown to be when initial performance was at its worst (Henderson Sabes and Sweetow, 2007; Ferguson et al., 2014), we would expect more clinically relevant benefit from music training for more of the hearing impaired. On the other hand, the exact opposite could also be the case, in which people with significant hearing issues will not benefit at all because they cannot really get involved in music interventions. This should be investigated in future studies.

Regarding our outcomes four main findings will be discussed: First, the left-sided improvement was greater in PP than in MC. Second, SIN mainly improved on the left, but not on the right side. Third, the German sample achieved better SRTs in comparison to the Swiss sample. And last, women showed an advantage of baseline SIN as well as a stronger improvement over time than men.

### SIN Improves Particularly by Instrumental Music Participation

We found that both PP and MC improved in SIN for binaural conditions. In other words, also a physically passive music intervention may induce beneficial auditory speech processing. It must be noted, however, that our study design does not include a passive control group, and therefore we cannot quantify the extent to which the general improvement over time was due to retest effects. This issue is further discussed in the limitations. A general effect on the left ear, however, is only present following *instrumental* music participation. This may be explained by the additional incorporation of the motor system during the learning process which may strengthen auditory-motor connections (Fleming et al., 2019; Zendel et al., 2019). According to this argument, choir singing and vocal training should be ideally suited to improve SIN through the activation of the auditory-vocal system. This hypothesis was confirmed experimentally in the study of Dubinsky et al. (2019). However, due to overlapping brain networks during instrument playing and singing, including primary motor (with larynx area), dorsal pre-motor and supplementary motor cortices (Segado et al., 2018), benefits in SIN are not exclusively induced by singing. A whole body of literature exists describing functional and structural adaptations of the auditory system due to musical training (Gaser and Schlaug, 2003; Herholz and Zatorre, 2012; Oechslin et al., 2013). It is evident that musical activities share many brain structures which are active in both speech processing and during SIN tasks, including the auditory cortex and premotor areas (see Coffey et al., 2017). Due to that, it is plausible that improvements in PP in the present study are also based on a refined auditory-vocal network. However, it remains to be clarified why this would only bring a SIN advantage for the left ear and, according to our results, cannot be generalized to all hearing conditions.

A multitude of studies have demonstrated that multimodal training promotes more effective learning than unimodal training (Lappe et al., 2008; Vongpaisal et al., 2016). For example, a short-term intervention study with 23 non-musicians Lappe et al. (2008) showed that multimodal sensorimotor-auditory training induces greater neuroplasticity in the auditory cortex than auditory training alone – and this was especially pronounced in the right hemisphere (related to the next discussion point). Additionally, *making* music (e.g., playing the piano) is, in comparison to mere music listening, highly connected to intensive goal-directed training, conditions of high arousal and strong emotional experiences and, therefore, complies with conditions of adaptive brain plasticity (Patel, 2011; Altenmüller and Furuya, 2017). An alternative but not mutually exclusive explanation is that active music interventions may lead to beneficial effects in executive functions (Schellenberg, 2004; Bugos et al., 2007; Hanna-Pladdy and MacKay, 2011; Seinfeld et al., 2013; Bugos and Kochar, 2017) which may contribute to SIN performance.

### SIN Improves Especially on the Left Side

Beside morphological asymmetries (Marie et al., 2015), also functional differences between the left and right auditory cortex seem to exist (for an overview see Zatorre and Zarate, 2012). As suggested already by Milner (1962) and Kimura (1964) on the basis of lesion and behavioral studies, later investigations using positron emission tomography (PET) and (f)MRI corroborated a clear hemispheric specialization in auditory functions: tasks which require a high temporal resolution are predominantly processed in the left auditory cortex (Zatorre, 2001; Hyde et al., 2008; Warrier et al., 2009), whereas the right auditory cortex is particularly involved in pitch perception (Zatorre et al., 1992; Zatorre, 2001). The only weak–moderate correlations between monaural conditions (Table 4) may indicate an independence concerning different underlying functional processes. This finding underlines the hypothesis of functional lateralization of auditory cortices. Another outcome also strengthens this dichotomy: PP showed an improvement of SIN when auditory stimuli were presented to the left, but not to the right ear.

Knowing that after neural encoding of acoustic features in the brainstem (Bidelman and Krishnan, 2010; Song et al., 2012), monaurally presented auditory stimuli project predominantly (via the superior olivary complex) to the contralateral auditory cortices (Suzuki et al., 2002), we hypothesize that SIN may have benefited from an improvement of more right-lateralized frequency discrimination following musical training (Magne et al., 2006; Moreno et al., 2009; Zendel and Alain, 2014; Du and Zatorre, 2017; Bianchi et al., 2019). This hypothesis is supported by literature showing a significant correlation of frequency discrimination with speech recognition or SIN (Parbery-Clark et al., 2009; Gfeller et al., 2012). Furthermore, with a 10-week study of choir singing and vocal training, Dubinsky et al. (2019) showed that training-related improvements in SIN were mediated by enhanced pitch discrimination. The authors also showed that the strength of the neural representation of pitch marginally moderated the relationship between SIN and pitch discrimination. Past neuroimaging studies demonstrated that music-driven plasticity in auditory regions are commonly right-lateralized: In these studies learning to play the piano induced significant structural brain changes in the right primary auditory region (Hyde et al., 2009), increased the response to speech in right temporal (superior/middle temporal gyrus) cortical regions (Fleming et al., 2019) and enlarged elicited musically mismatch negativity (MMNm), in particular from the right auditory cortex (Lappe et al., 2008).

In addition to spectral information, it could be argued that SIN-critical temporal discrimination may also improve by musical training (Rammsayer and Altenmüller, 2006; Kumar et al., 2016). Hence, according to the functional asymmetry of auditory cortices, improvements should also be expected for the right ear. Indeed it is plausible that temporal aspects are important for SIN and the absent general effect for the right ear may therefore be contradictory. One explanation might be that the intervention was too short to induce beneficial temporal effects; another, that fine-grained temporal resolution is—at least in certain languages – not as crucial for SIN as spectral factors. For example, Vermeire et al. (2016) showed that SIN is not correlated to gap-detection ability^2^ in a Dutch population and in a study by Hoover et al. (2015) only one out of two gap-detection tests could significantly explain variance in English SIN. It seems likely that different types of languages entail different demands on certain perceptual abilities and prosodic sensitivity. A fundamental characteristic to subdivide languages relies on their rhythmic division of time. In stress-timed languages (for example German) the duration between two stressed syllables is equal whereas in syllable-timed languages (for example French) the duration of every syllable is equal (Nespor et al., 2011). In other words, in comparison to German, French has less variability in vocalic duration which may render timing cues less important for word segmentation in continuous speech (Jun, 2014). This may be reflected in the correlation matrix of SIN (Table 4). The French Matrix Test showed a high correlation between the left and binaural condition (*r* = 0.87) and a weak correlation between the binaural and the right condition (*r* = 0.39), which may indicate a rather strong dependence on pitch cues (processed in the right auditory cortex) to understand French binaurally, i.e., in daily life. In the German version binaural conditions correlated moderately with both left and right conditions (*r* = 0.67 and *r* = 0.57, respectively), making also temporal resolution (processed in the left auditory cortex) important for understanding speech. Thus, the reported improvement of right SIN in the German sample would be expected. In other words, although all participants may have improved in temporal resolution, this improvement only significantly impacted SIN in German subjects. Future research should focus on further disentangling the contribution of temporal and spectral discrimination to SIN in different languages.

### German SRT Is Lower Than Swiss SRT

In all conditions we measured an approximately 2 dB lower SRT in the German versus Swiss sample. This difference probably cannot be explained solely by the sex difference of speakers used in the tests (male German speaker; female French speaker). Perceptual differences can appear due to the speaker’s sex, as shown for the German Matrix Test with a 2.3 dB difference between the male and female version (Wagener et al., 1999a; Wagener et al., 2014). However, in these studies the advantage was found in conditions with a female speaker. Hence, we could expect an even greater difference between German and Swiss samples if a German female speaker would have been used. A more likely explanation is a potential language effect (for a review see Kollmeier et al., 2015). For example, Hochmuth et al. (2015) showed differences of SRT across languages with the lowest score obtained in Russian (−10.2 dB), followed by Polish (−9.4 dB), German (−7.4 dB), and Spanish versions (−7.2 dB). The authors attribute these differences mainly due to spectral differences and masking effectiveness. The same reason may explain the divergence between the Hanover and Geneva participants in this study.

### Women but Not Men Show Improvement Over Time

Modeling binaural and left SIN revealed a substantial effect of sex and sex-by-time interaction, both with an advantage for women. That men are more affected by hearing loss than women is consistent with the literature (Agrawal et al., 2008; Moore et al., 2014). For example, Agrawal et al. (2008) found 2.4- and 2-fold higher odds of bilateral and unilateral hearing loss at speech frequencies in men compared to women. Although in this study hearing impairment was measured by pure-tone average (PTA) this method is highly correlated with SIN (Helfer and Wilber, 1990; Picard et al., 1999).

A common explanation for sex differences in hearing ability is based on the higher lifetime noise exposure in men (Dalton et al., 2001; Agrawal et al., 2008). In addition, the advantages of women in verbal skills and especially in verbal fluency (Hyde, 2014) may also contribute to SIN (but see Classon et al., 2014) and may be responsible for sex-specific behavioral differences. However, even if this might explain the sex differences we found at baseline, the question of why women and men progress differently *over time* remains to be solved.

A continuously emerging research interest, particularly in the field of sports science, focuses on sex-specific effects of interventions. In a meta-analysis with 39 included RCTs Barha et al. (2017) investigated sex differences in exercise efficacy to improve executive functions. The results revealed that all types of exercises^3^ were associated with larger effect sizes in studies with a higher percentage of female participants. Therewith, they could replicate a former meta-analysis comprising 18 RCTs showing larger aerobic training-related cognitive benefits for women than men (Colcombe and Kramer, 2003). Both studies may be in conflict with a more recent meta-analysis including 80 RCTs showing *less* general exercise effectiveness in women (Ludyga et al., 2020). However, subgroup analysis revealed that these differences in effect sizes were absent in low to moderate exercise intensities. Only in rather intensive exercise programs men derive more cognitive benefit than women. Furthermore, the authors could reveal a significant sex-specific exercise type-response relation indicating that female participants improve less in all exercise types, except for coordinative training. Clearly, practicing the piano places high demands on coordinative abilities, but on the other hand it is a physically low-intensive activity (Iñesta et al., 2008). In that the results of the mentioned meta-analyses fit to our results. The reasons for this sex-specific efficacy, however, are still to be found out but may be explained by the role of sex steroid hormones in neuroplasticity. A detailed discussion on this, however, is beyond the scope of the present paper and interested readers are referred to Barha et al. (2017) and Gurvich et al. (2018). For future research conducting a meta-analysis with musical interventions would be worthwhile to examine whether sex-specific efficacy on cognitive improvement also holds in the field of music.

### Strengths and Limitations

With 156 very carefully selected subjects with normal hearing and minimal musical experience we could presume a causal relationship between musical training and SIN. The argument that improvements of both groups derive from practice effects of the task itself is an important consideration as practice effects of the Matrix Test are well-known (Wagener et al., 1999a). However, the fact that benefits are side-specific and predominantly found on the left ear renders this argument unlikely to be true. In addition, the 6-month period between both test points was most likely long enough to significantly reduce participants’ recall ability. And finally, during all conditions we used different sets of sentences. Conclusively, we found it unlikely that retest effects are essentially attributable to the observed gain in SIN.

One limitation is that audiometric measurements (e.g., PTA testing) did not provide information on peripheral hearing. Although we excluded participants who reported suffering from hearing problems or wearing a hearing aid we cannot rule out that the participants had mild or moderate hearing loss. However, as a marker for peripheral hearing we included the intelligibility score in the statistical models. This variable captured the percentage of words which were correctly understood at 65 dB without background noise. In all models intelligibility predicted SIN and contributed in explaining variance. It should be noted nonetheless, that the central point of RCTs is the analysis of change and we were mainly interested in the development of SIN over time. Although PTA may explain baseline differences it struggles explaining the differences of progress (for example between men and women). Please also note that we did not find any baseline differences in SRT between PP and MC in any model.

A total of 26 teachers were recruited from local universities to hold the music courses. Although the teachers pursued the same goals and teaching principles, deviations in the curricula may have led to different study results among participants and make replication of the intervention difficult.

Group size is used as a proxy for training intensity (Clarke et al., 2017). Small groups may enable more individualized and intensive lessons and thus may lead to higher musical achievements. Since PP was taught in dyads and MC in small groups of 4–7 subjects, Time-by-Group interactions may be influenced by the group size. The effectiveness of group size is a poorly explored topic in music intervention research and remains a matter of future experiments. Some evidence comes from Jackson (1980), who found no differences in individual piano achievement within classes of two, four, six, eight, and twelve non-musicians. Clarke et al. (2017) addressed this topic in the field of mathematics and carried out an RCT in which, similar to our study, two-student groups (120 students) were compared with five-student groups (295 students). Their results also showed no significant differences in student achievement between both conditions. This finding could be replicated by Doabler et al. (2019) and confirmed in reading ability in older students (Vaughn et al., 2010). These findings indicate that smaller groups are not necessarily associated with better learning success and underline the potential of students interacting with and learning from each other (peer learning).

Further limitations include the only occasional monitoring of homework and the courses to assess practice quality and intervention fidelity, and, finally, the knowledge of the testers about the group membership of the participants. However, due to the design of the Matrix Test, it is unlikely that this knowledge biased the subjects’ results.

## Conclusion

The present study demonstrates that musical training, and especially playing an instrument, can counteract the age-related decline of SIN. The mechanisms of SIN enhancement due to musical activity are yet not clear. One explanation is that musical training enhances auditory processing (e.g., spectrotemporal discrimination). This may facilitate the formation of auditory objects — a skill which can be transferred to the domain of speech. Another explanation is that musical training or learning to play an instrument improves cognitive functions (e.g., inhibition, attention, and working memory) which may support SIN. Regarding the results, musical engagement should be considered as an auditory rehabilitation strategy in hearing loss and communication problems.

## Data Availability Statement

The raw data supporting the conclusions of this article will be made available by the authors, without undue reservation.

## Ethics Statement

The studies involving human participants were reviewed and approved by the Research Ethics Review Committee of Leibniz University Hanover and the Ethics Committee of Hannover Medical School (number 3604-2017) as well as the Cantonal Ethics Committee Geneva (number 2016-02224). The patients/participants provided their written informed consent to participate in this study.

## Author Contributions

FW wrote the initial draft of this manuscript. DM, FW, and LA acquired the data. FW and MG performed the statistical analysis. CJ and EA wrote the grant proposal submitted to the DFG (Deutsche Forschungsgemeinschaft) and SNSF (Swiss National Science Foundation). MK and TK gave detailed input to the grant application. All authors critically reviewed, revised the article, and read and approved the submitted manuscript.

## Conflict of Interest

The authors declare that the research was conducted in the absence of any commercial or financial relationships that could be construed as a potential conflict of interest.

## Footnotes

1 The credible interval describes the uncertainty and is roughly equivalent to the frequentist confidence interval, although their definition and interpretation are very different. Bayesian interference is not based on statistical significance. Instead it returns a *distribution* of possible effect values, which is called the “posterior.” The credible interval is the range of the posterior containing a particular percentage of probable effect values (here: 95%; for further information see Makowski et al., 2019).

2 The ability to detect silent periods within auditory stimuli is measured by gap-detection tests and relates to temporal discrimination.

3 In their study the authors distinguished between aerobic training, resistance training and multimodal training.

## Abbreviations

CI: Credible interval
SIN: Speech in noise
SRT: Speech reception threshold
PP: Playing piano group
MC: Musical culture group
RCT: Randomized controlled trial.
