# Supplementary material for: Improved Speech in Noise Perception in the Elderly After 6 Months of Musical Instruction
Source: Front Neurosci. 2021 Jul 9;15:696240. doi: 10.3389/fnins.2021.696240 (PMC8299120; doi:10.3389/fnins.2021.696240)
Supplement: Supplementary file 1 [file Data_Sheet_1.PDF]

First 6-month curriculum for Musical Culture

Organization of the courses:
- In between planned courses, elective courses were offered regularly
- Participants could regularly present a topic of their choice for ~10 minutes

The support material consists of extracts from textbooks, internet pages, documentaries, radio broadcasts and presentations (for example, “TED talks” (ted.com)) and links for listening mainly of YouTube videos and audio material. Internet links and other materials for the upcoming course are provided in the week preceding the lessons. Homework (30 min/day) involves resuming the course, listening to different musical pieces, reading texts and preparing presentations. Participants are continuously encouraged to express why he/she appreciates, or not, particular pieces or styles of music.

During the highly interactive courses, the teacher explains the structure of the pieces, the role of the different instruments and voices and affective aspects of the pieces and develops a structured way of listening (“auditory analysis”) among the participants.

1st Section

- Introduction: round table and presentation of the program
    - Presentation of the main goal of the course: active music listening and analysis
- Definitions of music
- The role of music the role of music throughout the world and history
- When did music come about? Prehistoric Music, Music of the Early Cultures
- Music in ancient Europe

2nd Section

- Music in the middle Ages – the development of polyphony
- Renaissance music
- Baroque music I: The emergence of forms (opera, concerto, etc.)
- Baroque music II: J.S. Bach
- Historically informed performance practice

3rd Section

- Introduction to the acoustics and physics of musical instruments; hearing
- Wind instruments: woodwinds, brass (musical examples)
- Percussion instruments & keyboard instruments (musical examples)
- The human voice (musical examples)
- String instruments (musical examples)

4th Section

- Classical period I: Haydn, Mozart, early Beethoven
    - Excerpts from : “My life with Mozart”, by Eric-Emmanuel Schmitt
- Classical period II: Forms: ouverture, symphony, solo concerto, string quartet, sonata …
- Romantism I: Beethoven, Schubert, Schumann, Brahms …
- Romantism II: Wagner, Liszt, Chopin
- Women in music history

First 6-month curriculum for Playing Piano

Each participant received an electronic piano, adjustable stool, and headphones for homework[1].

Courses: In the beginning, the participants performed imitation and listening exercises guided by the teacher. Most of them were playful and allowed the participants to familiarize themselves with the keyboard and adopt a correct and relaxed body posture.

The three essential components of the courses are:

1) (~10 min) Warming-up, posture exercises, exploring the keyboard
2) (~40 min) Learning to play the piano, alone and together, with and without accompaniment, first with one hand (alternating right and left), then with both hands; first only by ear, later on also by reading a score; improvisation; constant reminders of correct body posture
3) (~10 min) Precise homework instructions

The pillars for learning to play the piano are: 1) **listening**, 2) **sensory and motor skills**, 3) **rhythm**, 4) producing **musical idiom** (through listening or through score reading), 5) experiencing **pleasure**, 6) **creativity** (within a musical piece or by improvisation)

Month 1

Examples of exercises:

- Dissociate white and black keys; twins and triplets; the seven full octaves; high vs. low; soft vs. loud
- Hit a key with different strengths; listen carefully, "Genie[2]","Kangaroo[3]", and "Feeling dust[4]" exercises with variations; play motives, for example, the "Telecom-Motive" (CCCEC) or Beethoven 5th (EEEC) in all octaves
- Initiation to improvisation. Example: improvise pentatonic melodies on black keys; dialogical-improvising (question-answer);
- Include the pedal right away
- Singing and playing alternately; translating rhythms into spoken text; playing silently on top of the piano with correct hand position; clapping, singing or walking within a certain rhythm

Homework:
Correct posture: sitting; distance to the keyboard; leg position; finger and hand position, Genie, Kangaroo exercises (these exercises should remain in the exercise program for a long time and ultimately be practiced with both hands and all fingers); improvisation on black or

[1] Yamaha Germany & Yamaha Switzerland generously offered the electronic pianos (Yamaha P-45 B).

[2] A self-selected key in the lower middle position is initially hit as softly as possible while holding the pedal. This is followed by calm repetitions of notes with a crescendo that is as finely graduated as possible up to a justifiable fortissimo (from “not yet sound” to “no longer sound”) and back again.

[3] Here, all keys with identical note names should be hit in a steady but constant meter, one octave apart, from bottom to top and back. The left hand plays on the left half of the keyboard, the right hand on the right half. This exercise can be varied and expanded in many ways: with the pedal; with different fingers; with additional tones (intervals smaller than an octave).

[4] With an upright posture while sitting at the piano, use all ten fingertips feel the imagined dust alternately on the level of the keys and on the level of the music stand. The movement from one level to the other should be elegant and characterized by smoothness in the shoulders, elbows and wrists. Also with eyes closed.

white keys; try to play a simple song by ear on the piano that was learned in class but also new ones, even with eyes closed.

Month 2-4

New exercises: "dolphin[5]", "mountain[6]"

All exercises (genie, kangaroo, feeling dust, dolphin, mountain) are repeated regularly throughout the first 6 months of training with many variations. By means of that, body posture and freedom of movement are continuously trained. This also holds for motive imitation and playing by imitation (by ear).

Regular playing with eyes closed.

The individual piano teachers introduced music reading progressively using a method specifically developed for our elderly population based on Jens Schlichting's "Piano Prima Vista" (Internote GmbH Musikverlag 2013), and the Hall Leonard piano method for adults volume I (ISBN: 9789043134378). Both methods exist in German and French.

Music reading was introduced later or earlier by individual piano teachers, some only taught by listening and imitation during this period.

Other materials:
- Simple pieces of the "Jugend-album für Klavier" by Manfred Schmitz (ISBN: 9789043134378)
- "A dozen a Day", volume 1 (ISBN: 9780711954311);
- A simple arrangement of "Ode to joy" (score provided below)
- Improvisation using different triggers: mood, drawings, a motive.

Homework:
All exercises, new and old pieces trained during the courses, improvisation.

Month 5-6

All exercises (genie, kangaroo, feeling dust, dolphin, mountain) are repeated regularly throughout the first 6 months of training with many variations. By that means, body posture and freedom of movement are continuously trained. This also holds for motive imitation and playing by imitation (by ear).

Regular playing with eyes closed.

Other materials:
- Jens Schlichting's "Piano Prima Vista" (Internote GmbH Musikverlag 2013)
- Transcriptions of favorite pieces of the participants, arranged by the music teachers (Amélie Poulenc (Yann Tiersen); Dvorak Symphony "From the New World", etc.)

---

[5] Variation of the kangaroo: the selected notes are played legato alternately with both hands – initially again at octave intervals, so that one hand "dives" and the other "flies". Here, too, the octaves can be filled in later, so that, for example, broken chords are created.

[6] Each finger is placed in its theoretically ideal position on the key (or a solid surface), so that the knuckle forms the highest point with fingers 2 to 5 and the underside of the wrist and fingertips are at approximately the same height. The fingers are stable and round. In this position, the finger is now stressed for several seconds, then: conscious, quick and complete loosening.

- More complicated pieces of the “Jugend-album für Klavier” by Manfred Schmitz (ISBN: 9789043134378)
- Hall Leonard piano method for adults, volume II (ISBN: 9789043152037).

Learning some basics of music theory, tonality, half and whole tones, chord progressions.

Playing in front of the other participant on a voluntary basis.

Homework:
All exercises, new and old pieces trained during the courses, improvisation.

« Hymne à la joie » / *An die Freude*

Symphonie no. 9 - 4e mouvement

(v3) - op. 125

♪ = 112

Ludwig van Beethoven (1770 - 1827)

arr. Xavier Favier

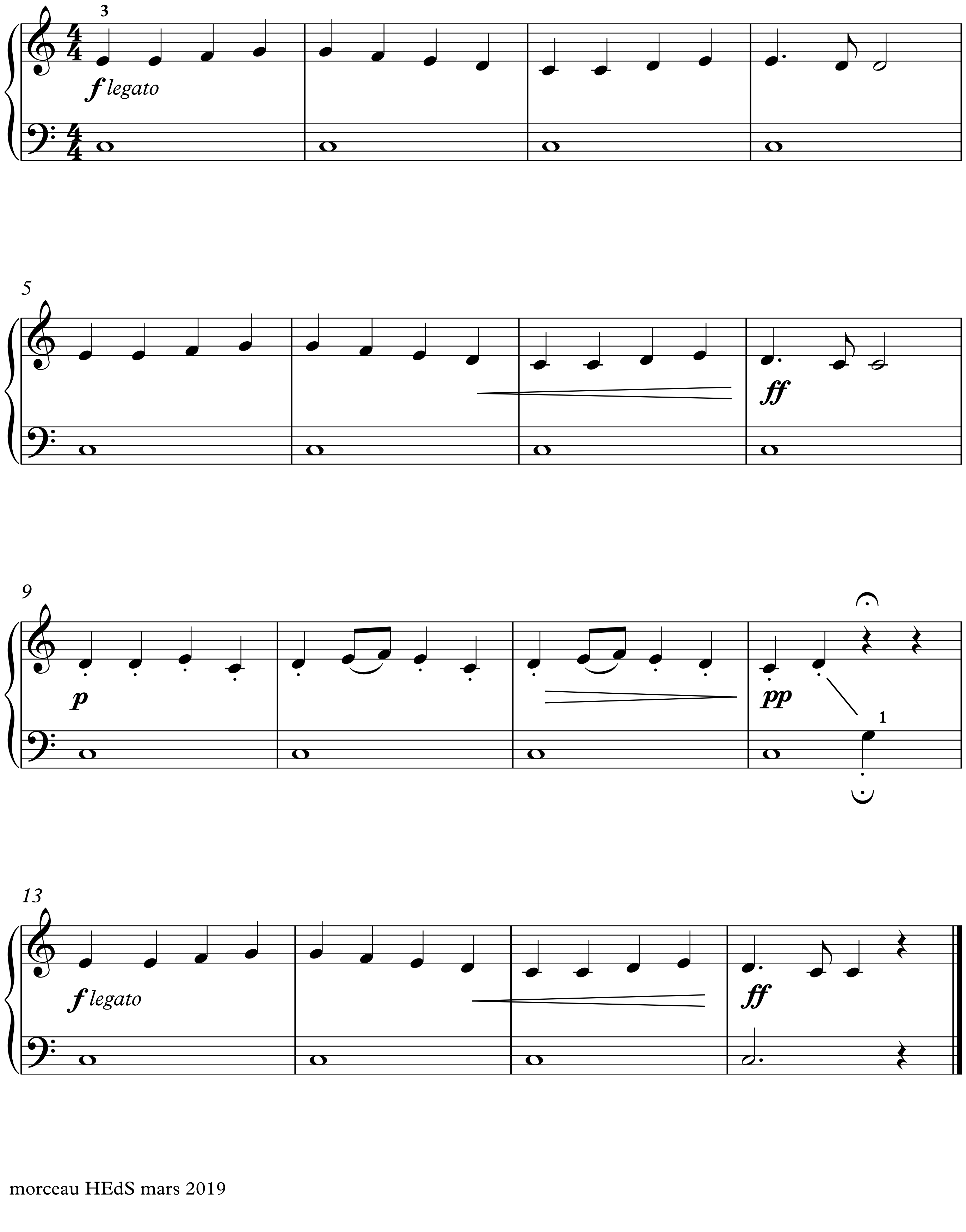

morceau HEdS mars 2019
